# Bacterial Persister Cells as Evolutionary Catalysts of Antibiotic Resistance: Mechanisms, Clinical Implications, and Therapeutic Strategies

**DOI:** 10.3390/antibiotics15060526

**Published:** 2026-05-22

**Authors:** Tae-Jong Kim

**Affiliations:** Department of Forest Products and Biotechnology, Kookmin University, Seoul 02707, Republic of Korea; bigbell@kookmin.ac.kr

**Keywords:** antibiotic resistance evolution, antibiotic tolerance, antipersister strategies, bacterial persister cells, biofilms, phenotypic heterogeneity, stress response networks

## Abstract

Antibiotic resistance is a growing global health threat. However, its evolution cannot be fully understood without considering antibiotic tolerance and persistence. Persister cells are phenotypic variants that survive lethal antibiotic exposure without heritable resistance, primarily through growth arrest, metabolic slowdown, and stress-adaptive states. Although persistence has been viewed as a transient survival phenomenon, increasing evidence suggests that it may also have a genetic basis by preserving populations during antibiotic-induced bottlenecks and enabling regrowth, mutation, and selection under certain conditions. This review examines the molecular mechanisms underlying persister formation, including toxin–antitoxin systems, stringent-response signaling, ATP depletion, translational arrest, and stress-response networks. We discuss how persistence contributes to antibiotic tolerance in biofilms, host environments, and recurrent infections, and how repeated antibiotic exposure may promote stepwise evolution from phenotypic survival to stable resistance in specific contexts. Evidence from experimental evolution, clinical observations, and system-level analyses supports a potential but context-dependent link between persistence and resistance. We also highlight therapeutic strategies targeting persister cells, including antipersister compounds, metabolic activation, combination therapies, bacteriophages, and alternative approaches. Finally, we outline future research directions, emphasizing single-cell technologies, systems biology, longitudinal clinical studies, and evolution-informed treatment design. A comprehensive understanding of persistence and its evolutionary implications is essential for improving treatment efficacy and limiting the emergence of long-term antibiotic resistance.

## 1. Introduction

Antibiotic resistance is a major global health challenge associated with increased mortality, prolonged hospitalization, and rising healthcare costs [1,2]. Although its clinical importance is well established, the processes that enable bacterial survival during antibiotic exposure are not fully explained by genetic resistance alone. This gap has increased interest in non-heritable survival phenotypes, particularly persistence and tolerance, as potential contributors to treatment failure and resistance emergence [3,4]. These challenges are further exacerbated by the limited development of new antibiotics, which further underscores the need for a deeper understanding of bacterial survival strategies that reduce antibiotic efficacy and lead to recurrent infections [5,6].

Under antibiotic exposure, bacterial populations experience strong selective conditions that can favor survival of rare variants or phenotypic subpopulations capable of withstanding treatment [7,8,9]. However, survival during early antibiotic exposure is not always explained by pre-existing genetic resistance, highlighting the importance of transient physiological states that permit survival before stable resistance emerges [4,10]. Cells that survive antibiotic exposure may contribute to subsequent resistance emergence under certain conditions. Phenotypic heterogeneity within genetically similar bacterial populations can strongly influence survival during antibiotic exposure. A representative example is the persister cell, a rare phenotypic variant that survives lethal antibiotic treatment without heritable resistance [3]. Because persister cells adopt low-metabolic or growth-arrested states, they are less vulnerable to antibiotics targeting active cellular processes. Under conditions involving repeated exposure to antibiotics, such as chronic infections, their survival may preserve a subpopulation from which later adaptive change can occur under favorable conditions. These observations suggest that persistence may have broader implications than short-term treatment survival alone. However, the extent to which persistence contributes to the emergence of resistance still varies depending on the situation, and the relationship among persistence, tolerance, and resistance remains a subject of debate across various bacterial systems [4,10].

This review examines the relationship between bacterial persistence and antibiotic resistance, with a focus on how persistence may influence survival, adaptation, and treatment outcomes under antibiotic stress. Through an integrated perspective combining molecular mechanisms, evolutionary dynamics, and clinical relevance, we aim to critically evaluate current evidence rather than assume a uniform persistence-to-resistance pathway.

## 2. Concept and Historical Background of Bacterial Persister Cells

The concept of persister cells among bacteria arose from resistance-related observations that could not be easily explained by classical genetics. In 1944, Joseph Bigger was the first to report a small subpopulation of *Staphylococcus aureus* cells that survived exposure to penicillin despite being susceptible at a gene level. These cells were not mutants, and their progeny remained susceptible when reexposed to the same antibiotic after subculture. Bigger named these “persisters”, recognizing that bacterial subpopulations exhibit intrinsic heterogeneity in antimicrobial stress response. These early observations challenged the prevailing view at that time that equated antibiotic survival with genetic resistance, and laid a conceptual groundwork for understanding persistence as a phenotypic phenomenon [11,12,13].

For decades, persister cells have been regarded as a microbiological curiosity with limited clinical significance. The dominant paradigm in antimicrobial research involves genetically inherited resistance mechanisms—mutations in drug targets, production of antibiotic-degrading enzymes, reduced intracellular drug accumulation, and so forth. Persister cells were often considered a transient, exceptional phenomenon because they do not harbor stable resistance-conferring mutations. However, advances in single-cell DNA techniques and population biology have revealed that phenotypic heterogeneity among genetic clones is inherent to bacterial communities, which can manifest as variations in metabolic activity, growth rate, stress response, and antibiotic susceptibility. In this context, persister cells are understood as a subpopulation with a distinct phenotype capable of surviving lethal antibiotic exposure without genetic alterations [3,12,13].

A critical characteristic of persister cells is their transient and reversible tolerance to antibiotics [3,14]. Unlike resistant mutants, persister cells typically exhibit suppressed metabolism or growth arrest, sometimes entering a dormancy-like state, making them less vulnerable to drugs that target active cell metabolisms. Once the antibiotics are removed, they can regrow and reconstitute a population with the same susceptibility as the original culture. Therefore, persistence is best understood as a reversible phenotypic state of antibiotic tolerance, distinct from heritable genetic resistance [3]. Such distinction is vital for conceptual clarity and clinical interpretation. Resistance alters the minimum inhibitory concentration (MIC) of an antibiotic for a specific strain, whereas persistence affects the bactericidal ratio without necessarily changing the MIC. In a bactericidal curve-plotting experiment, a resistant population grew continuously even in the presence of an antibiotic, whereas one containing persisters exhibited a biphasic bactericidal pattern, the number of surviving cells initially declines sharply, followed by a plateau corresponding to the surviving persister fraction [3,14].

Distinguishing between persistence and resistance offers important insights for interpreting treatment outcomes. Resistance can be readily identified through routine susceptibility assessments. However, persister cells are generally rare and do not grow in the presence of antibiotics under MIC-testing conditions. Hence, it may not be detected during diagnosis. Nevertheless, they can endure prolonged antibiotic exposure and may cause infection recurrence once selection conditions are removed. Therefore, treatment failure or recurrence can occur even when laboratory tests show complete susceptibility of the pathogen. This phenomenon highlights the limitations of existing diagnostic systems based solely on genetic resistance [3,12,13].

The molecular mechanisms underlying persistence have gradually been elucidated over the past two decades. Contributing factors include toxin–antitoxin (TA) systems, stringent responses mediated by (p)ppGpp (guanosine tetraphosphate and pentaphosphate), metabolic repression, ATP depletion, ribosomal inactivation, and global stress-response pathways. Crucially, no single mechanism universally defines persistence; rather, diverse physiological pathways converge toward a common outcome—reduced cell activity and antibiotic tolerance [13,15]. Stochastic fluctuations in gene expression can spontaneously generate a quiescent-like state, while environmental stimuli, such as nutrient deprivation, oxidative stress, and antibiotic exposure, can also induce persister formation. This mechanistic diversity supports the notion that persistence is not a strain-specific trait but rather an emergent property of bacterial populations exposed to fluctuating environments.

The clinical significance of persister cells in chronic and recurrent infections has gained recognition, including those associated with biofilms, medical devices, chronic wounds, and the lungs of cystic fibrosis patients, which often exhibit enhanced antibiotic resistance. Nutrient and O_2_ concentration gradients within biofilms promote metabolic heterogeneity and create an environment favorable for persister formation. Similarly, upon exposure to host-derived stress, intracellular pathogens can transform into a dormant state, enabling survival despite antibiotic treatment. Diseases like tuberculosis require prolonged treatment to eliminate actively proliferating bacteria and slow-growing or dormant subpopulations. These clinical observations demonstrate that persistence is not a laboratory construct but a biologically and medically significant phenomenon [12,13].

Persistence carries not only immediate clinical implications but also deeper evolutionary significance. Persister cells maintain population continuity by enabling a portion of it to survive lethal antibiotic exposure, potentially allowing subsequent adaptive changes or acquisition of resistance determinants, even though their resistance is not genetically encoded [4,10]. In conditions requiring repeated antibiotic treatment, such as chronic infections, this dynamic recurrently induces bottlenecks while preserving a reservoir of viable cells. Over time, this cycle can promote gradual accumulation and fixation of resistance-conferring mutations. Thus, persistence, once viewed as a transient survival phenomenon, can involve long-term evolutionary pathways, a concept reflecting a paradigm shift in microbiology. Initially described as an abnormal survival-associated phenomenon, it was later clarified as resistance, which is not genetically encoded and is now understood as an evolutionary strategy integrating phenotypic heterogeneity and population dynamics. Such a definition moves beyond traditional binary classification of bacteria as either susceptible or resistant, emphasizing instead a spectrum of physiological states that shape therapeutic outcomes and evolutionary potential. Therefore, understanding the conceptual and historical basis of persistence is essential for integrating a comprehensive evolutionary framework of antimicrobial resistance with phenotypic resistance [3,4,13].

## 3. Molecular Mechanisms Underlying Persister Cell Formation

Although multiple molecular mechanisms have been implicated in persister formation, their relative contributions remain heterogeneous. Some mechanisms are supported by substantial experimental evidence across multiple systems, whereas others remain debated or context-dependent, and their roles may vary depending on bacterial species, environmental conditions, and experimental design.

### 3.1. Toxin–Antitoxin (TA) Systems

TA systems have been proposed as molecular modules associated with persister cell formation; however, their contribution remains controversial, with some studies reporting significant involvement while others find minimal or no effect depending on the system [15,16]. They are generally composed of a cognate toxin–antitoxin pair and are distributed on a wide range of bacterial chromosomes and plasmids [17,18]. Under normal growth conditions, the antitoxin neutralizes the toxin, whereas under stress, the degradation or functional loss of the antitoxin activates the toxin. Activated toxins inhibit essential cell processes, including translation, mRNA stability, and DNA replication, thereby arresting growth [18,19,20], shifting cells into a transient low-metabolic or growth-arrested state associated with antibiotic tolerance [15,19,20]. As many bactericidal antibiotics are most effective against actively growing cells, toxin-induced growth arrest can reduce their lethality. However, this state is reversible and distinct from a heritable resistance [15].

TA systems may contribute to phenotypic heterogeneity among clonal populations. Stochastic fluctuations in the toxin–antitoxin balance may generate distinct physiological states, thereby increasing the fraction of cells that can survive antibiotic exposure. Thus, TA systems may contribute to a bet-hedging-like survival strategy at the population level [15]. From an evolutionary perspective, TA-associated growth arrest may prolong survival under antibiotic stress, thereby providing additional time for the emergence or acquisition of adaptive resistance determinants; however, the long-term contribution of TA systems to resistance evolution remains incompletely resolved [15,16].

### 3.2. Stringent Response and (p)ppGpp Signaling

Such a stringent response is a major global regulatory mechanism that rapidly reprograms bacterial physiology under nutritional and environmental stress. The alarmone “(p)ppGpp” is a key mediator, whose levels are primarily controlled by enzymes belonging to the RelA/SpoT family [21,22]. When (p)ppGpp accumulates, it suppresses ribosome biogenesis and other growth-associated processes while promoting stress adaptation and survival functions [15,21]. Accordingly, cells shift from a growth to a survival-oriented state, and altered persister frequencies in strains with modified (p)ppGpp metabolism suggest that the stringent response represents an important regulatory axis associated with persistence, although its contribution may vary depending on context [15,22]. (p)ppGpp directly modulates RNA polymerase activity, leading to global transcriptional reprogramming, and also affects DNA replication, translation, and metabolic pathways.

In certain conditions, (p)ppGpp-based signaling may interact with TA systems and other stress-response pathways, in part through regulatory cascades that may activate protease Lon, lead to antitoxin degradation, and subsequently activate toxin [15]. Thus, the stringent response is best viewed not as an isolated mechanism but as part of an integrated regulatory network linked to metabolic slowdown, ATP depletion, ribosome modulation, and antibiotic tolerance [15,22,23]. This network-level modulation may enhance physiological heterogeneity within clonal populations, allowing a subset of cells to enter low-metabolic or deeply growth-arrested states [15,23,24].

From an evolutionary perspective, stringent-response-mediated growth limitation may provide population survival under antibiotic stress and increase the probability of subsequent adaptive evolution [22,25]. Recurrent antibiotic exposure can repeatedly activate survival programs associated with persistence, facilitating cycles of survival, regrowth, and reselection [15,25]. Although such dynamics may eventually favor the emergence of genetics-derived resistance, the contribution of (p)ppGpp signaling to its evolution is regarded as indirect and context dependent [22,23,25].

### 3.3. Energy Metabolism and ATP Depletion

Alterations in cell-level energy metabolism, particularly suppressed intracellular ATP levels, have emerged as an important physiological correlate and mechanistic contributor to persister cell formation [26,27,28,29]. Bacterial susceptibility to many antibiotics is closely linked to active cell processes, including DNA replication, transcription, translation, and cell wall synthesis, which require substantial energetic input [15,29]. By contrast, cells with reduced ATP levels have diminished biosynthetic activity and slowed growth, thereby limiting the effectiveness of antibiotics that target active physiological pathways. Consequently, low-energy states are strongly associated with transient antibiotic tolerance [15,26,27].

Experiments have demonstrated a pronounced relationship between intracellular ATP concentration and persister frequency [26,27,28,30]. Artificial ATP reduction through metabolic inhibition, nutrient limitation, or perturbation of respiratory activity can enhance the proportion of persister cells within a population, whereas metabolic stimulation often decreases persistence [26,27,28]. These findings suggest that ATP depletion is strongly associated with persister formation; however, whether it represents a driving mechanism or a downstream consequence of growth arrest remains unresolved and may vary across systems [26,27]. In this context, metabolic heterogeneity within bacterial populations generates a spectrum of energetic states, among which low-ATP levels are frequently observed in cells that survive antibiotic exposure, although this association does not necessarily imply a direct causal relationship [27,28,29].

In particular, energy depletion interacts with other regulatory networks implicated in persistence, including stringent-response signaling and translation-inhibitory programs [15,29]. Such interconnected pathways converge on a common physiological outcome: a reversible low-energy state characterized by growth arrest and heightened antibiotic tolerance [15,25,29]. Thus, declined ATP levels can be considered a recurring metabolic feature shared by multiple persister-forming routes rather than an isolated phenomenon [15,25,29].

Beyond its immediate impact on antibiotic tolerance, metabolic downregulation may also present evolutionary implications. By promoting survival during antibiotic-induced bottlenecks, low-energy persister states may prolong population survival and preserve opportunities for subsequent adaptive evolution [25,31,32]. Upon antibiotic withdrawal and metabolic reactivation, surviving cells can resume growth and re-enter later selection cycles [25,31,32]. Accordingly, metabolic heterogeneity and ATP depletion may indirectly induce conditions that favor the eventual emergence and selection of heritable antibiotic resistance [25,31,32].

### 3.4. Ribosome Modulation and Translational Arrest

The regulation of ribosome activity and translational arrest are one of key physiological features associated with the formation of persister cells [15,33,34,35,36]. Many bactericidal antibiotics exhibit maximal efficacy in actively growing cells, and the suppression of protein synthesis can reduce the activity of growth-associated targets [15,36]. When translation is reduced, metabolic activity declines, and cells can enter low-growth or quasidormant states, thereby limiting the lethality of antibiotics that depend on active biosynthesis and replication [15,36,37].

This effect is generally mediated by reversible regulatory mechanisms rather than permanent ribosomal modification [35,36]. Certain toxins from TA systems inhibit translation by cleaving mRNA, targeting tRNA, or interfering with ribosome-associated processes [33,34]. In addition, (p)ppGpp signaling suppresses ribosome biogenesis, whereas the associated hibernation factors promote the formation of inactive ribosomal complexes, such as 100S particles, during stress [15,35]. These processes converge on reduced translational capacity, a recurrent physiological feature of many persister states [15,33,34,35,36]. As this state is reversible, cells already in low-translation states may be preferentially enriched during antibiotic exposure and contribute to the persister fraction [35,36].

From an evolutionary perspective, ribosome modulation may contribute to short-term survival under antibiotic stress by limiting growth-associated processes, thereby maintaining opportunities for later adaptive evolution [36,37,38].

Collectively, these mechanisms also should not be interpreted as universally acting or independent drivers of persistence. Rather, they represent interconnected and context-dependent processes whose relative importance differs across bacterial systems, highlighting the need for cautious interpretation of mechanistic contributions.

### 3.5. Stress Response Networks

Persister cell formation is best understood as an integrated regulatory process comprising interconnected stress-response networks rather than a single molecular mechanism [13,15,39]. When bacteria encounter nutrient limitation, oxidative stress, DNA damage, or antibiotic exposure, they activate overlapping transcriptional and metabolic reprogramming programs that shift physiology from a growth to a survival-oriented mode [13,15,39,40]. One example is the SOS response, which promotes DNA repair and, in certain contexts, enhances mutation frequency via the induction of error-prone polymerases [41,42]. Responses to oxidative stress are also associated with antibiotic exposure and may help limit cell damage while contributing to physiological states linked to persistence [42,43]. Global regulators, such as the alternative sigma factor RpoS, integrate diverse stress cues and induce broad changes in gene expression patterns [40]. These systems intersect with (p)ppGpp signaling, TA modules, metabolic slowdown, and ribosome-associated growth arrest, thereby amplifying physiological heterogeneity within clones [39]. As a result, a subset of cells may enter growth-arrested or low-metabolic states and survive subsequent antibiotic exposure [13,39]. Because SOS activation can enhance mutation frequency under stress, response networks may influence short-term survival and the likelihood of later adaptive changes, positioning them at the critical persistence–resistance emergence interface [41,42,44].

Thus, persister formation is better regarded as a population-level physiological state induced by the synergistic action of multiple stress-response pathways rather than the effect of a single gene. By sustaining population survival through repeated antibiotic bottlenecks, these networks may preserve opportunities for subsequent adaptive diversification and resistance evolution.

For direct comparison of the major mechanisms proposed to underlie persister formation, a comparative summary is provided in Table 1, including their proposed roles, supporting evidence, studied organisms, and major limitations. As summarized in Table 1, these mechanisms should be interpreted as interconnected and context-dependent processes rather than universal or independent drivers of persistence.

In addition to classical stress-response pathways, bacterial gasotransmitter systems such as hydrogen sulfide (H_2_S) and nitric oxide (NO) have recently emerged as important contributors to antibiotic tolerance and persistence-associated survival [45,46]. These systems can protect bacteria from oxidative stress and antibiotic-induced damage through modulation of respiration, redox homeostasis, and stress signaling pathways, thereby enhancing survival under antimicrobial exposure. Recent evidence further suggests that metabolic defense pathways, including ribose-5-phosphate (R5P) biosynthesis, may also contribute to bacterial protection against antibiotic-induced stress and persistence-associated survival [47]. Accordingly, pharmacological disruption of H_2_S- or NO-mediated defense systems and related metabolic protective pathways has been proposed as a potential antipersister strategy.

## 4. Antibiotic Tolerance Is Mediated by Persister Cells

### 4.1. Temporary Antibiotic Survival Mechanisms

Persister cell survival under antibiotic exposure is primarily associated with physiological states such as low-metabolic activity, growth arrest, translational slowdown, and/or reduced energy availability. As many bactericidal antibiotics target cellular processes such as β-lactams for cell wall synthesis, fluoroquinolones for DNA replication, and aminoglycosides for protein synthesis, cells in which these are suppressed are relatively more resistant to antibiotics. Such physiological states may also reduce vulnerability to antibiotic-induced cell damage. Importantly, this state is reversible: once antibiotics are removed, persister cells can resume growth and division, and their progeny regain antibiotic susceptibility; such a phenomenon is a defining feature that distinguishes persistence from heritable resistance [3,12,48,49]. Throughout this section, persistence, tolerance, and resistance are treated as distinct phenomena: persistence refers to a transient, non-heritable state in a subpopulation; tolerance describes a population-level reduction in antibiotic killing without a change in minimum inhibitory concentration; and resistance denotes heritable genetic adaptations that enable growth in the presence of antibiotics.

### 4.2. Biofilms and Persistence

Biofilms represent a classic environment in which persister formation and antibiotic tolerance are prominent. Within biofilms, nutrient and O_2_ gradients, accumulation of metabolic byproducts, and increased cell density generate robust physiological heterogeneity, promoting slow-growing and hypometabolic subpopulations. These tolerant populations and persister subpopulations can contribute to repeated failures in treating biofilm-associated infections, although they represent distinct physiological states, including chronic lung or wound infections and those caused by contaminated medical devices. Cells within biofilms can regrow after antibiotic withdrawal, thereby promoting recurrence and maintaining selective pressure across repeated treatment cycles [50,51,52,53].

### 4.3. Persistence Induced in the Host Environment

Persister formation occurs not only in vitro but also within host environments, where immune pressure, nutrient limitation, acidic pH, hypoxia, and intracellular stresses can promote antibiotic tolerance. This phenomenon is particularly relevant for chronic infections, in which antibiotic concentrations used may fail to achieve sufficient activity and demonstrate limited efficacy against sluggish or inactive bacterial subpopulations. Under such conditions, surviving persister cells can serve as a reservoir for regrowth after treatment cessation [53,54,55,56,57].

## 5. From Persistence to Resistance: Evolutionary Connections

**Figure 1:**
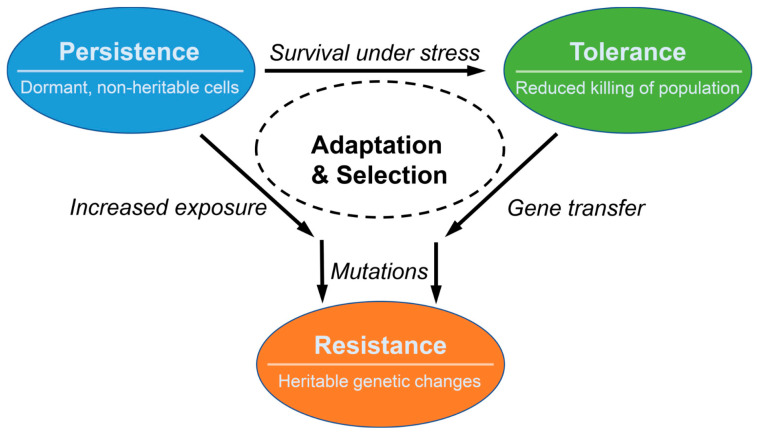
Conceptual model illustrating the context-dependent pathways linking persistence, tolerance, and resistance. Persistence represents a transient, non-heritable survival state in a subpopulation, whereas tolerance reflects reduced killing at the population level. Resistance arises from heritable genetic changes. These states may interact under certain conditions, but their relationships are not necessarily linear, sequential, or deterministic.

A conceptual overview of the proposed relationships among persistence, tolerance, and resistance is provided in Figure 1.

### 5.1. Increased Mutation Rates Under Stress

Antibiotic stress not only eliminates susceptible cells but can also reshape the genetic-level dynamics among bacterial populations by altering survivability and the opportunity for adaptive variation [4,9,10,41,58]. Under some stressful conditions, mutation rates can increase through the induction of error-prone DNA polymerases, altered DNA repair, and broader stress-responsive regulatory changes [9,41,59]. Such a phenomenon is commonly referred to as stress-induced or adaptive mutagenesis and is thought to increase the probability of adaptive genetic changes under adverse conditions.

Antibiotic exposure is a major trigger of such responses. For example, those that damage DNA can activate the SOS response, potentially increasing the probability of mutations through error-prone repair pathways in some contexts [41,60]. Oxidative stress and metabolic imbalance may also contribute to DNA damage and altered mutation-associated processes, thereby expanding genetic variability [9,41]. Although most such mutations are neutral or deleterious, rare beneficial variants that enhance antibiotic resistance can be rapidly enriched under selective conditions [9,10]. In this context, persister cells are particularly relevant because they preserve survival during antibiotic-induced bottlenecks, thereby allowing adaptive mutations to emerge and be selected during regrowth in certain contexts [4,10,58]. Repeated cycles of antibiotic exposure and recovery can therefore promote survival, regrowth, and reselection of more adaptable lineages.

Stress-induced regulatory systems, including the SOS response and other global pathways, influence survival physiology and the production of genetic variations [4,41,58,60]. Accordingly, antibiotics can act not only as bactericidal agents but also as selective environments that couple population-level bottlenecks with opportunities for adaptive diversification [4,10,58,59,60]. In this sense, stress-associated mutational processes represent a crucial mechanistic bridge linking persistence-associated survival to the subsequent emergence of genetic resistance.

### 5.2. Persistence as a Reservoir for Resistance Emergence

Persister cells do not carry heritable resistance, but act as a surviving reservoir that may contribute to the emergence and enrichment of tolerant lineages under certain conditions. During antibiotic treatment, most susceptible cells are eliminated, whereas persister cells survive in a transient low-metabolic state. This phenomenon may prevent population collapse and enable regrowth once selective pressure is relieved. By preserving population continuity across these bottlenecks, persisters maintain opportunities for mutation-based adaptation and, in suitable ecological settings, the acquisition of resistance determinants [4,10,31,44,61,62].

Following the decline or removal of antibiotic pressure, persister-derived survivors can resume proliferation. During this regrowth phase, spontaneous mutations can accumulate, and newly resistant offspring may be selected during subsequent treatment cycles [10,61,63]. This process supports the view that persistence may provide an initial survival advantage that, under certain conditions, can be associated with subsequent resistance evolution [4,10,31,61,63]. Persistence may also preserve opportunities for horizontal gene transfer and the subsequent fixation of acquired resistance determinants in appropriate environments; persister-associated reservoirs can promote the spread of resistance plasmids [4,31,44,62]. Accordingly, persistence is not only a physiological phenomenon but also an ecological and evolutionary condition that can create conditions under which resistance may emerge under repeated antibiotic selection [4,31,44,61,62].

Persistence should therefore not be viewed as opposed to resistance but rather as a phenotypic state that can, in some contexts, precede and contribute to resistance emergence, while alternative independent pathways to resistance are also well established [4,10,31,44,61,63]. From this viewpoint, persistence may contribute to therapy failure and, in some contexts, influence subsequent resistance development [4,31,44,61,62]. Importantly, the relationship between persistence and resistance is not universal and remains strongly context-dependent. While numerous studies suggest that persistence may facilitate resistance emergence under antibiotic pressure, resistance can also arise independently of persistence through the direct selection of pre-existing or newly generated resistant mutants. Furthermore, persistence does not necessarily increase evolutionary rates; in some systems, prolonged dormancy or reduced metabolic activity may limit opportunities for genetic change. These observations indicate that persistence represents one of several possible pathways contributing to resistance evolution rather than a deterministic or obligatory intermediate state.

### 5.3. Adaptive Evolution During Recurrent Antibiotic Exposure

As illustrated in Figure 1, these relationships should be interpreted as context-dependent interactions rather than a fixed stepwise pathway. In clinical settings, antibiotic exposure often occurs repeatedly or intermittently rather than as a single isolated event, generating recurrent cycles of killing and regrowth [4,10,32,63,64]. Such continuous reexposure induces periodic bottlenecks and recovery phases within bacterial populations, creating conditions favorable for adaptive evolution [4,10,32,63]. Each cycle removes large numbers of susceptible cells, whereas surviving tolerant or persister-derived ones can rebuild the population during the recovery phase [4,10,32,63]. Such a repetitive sweep–recovery dynamic continuously reshapes the bacterial community and may, in certain contexts, contribute to shifts from phenotypic survival toward stable genetically acquired resistance, although alternative evolutionary outcomes are also possible [4,10,63]. As discussed in Section 5.2, persistence-associated survival may influence subsequent adaptation; here, we focus specifically on how repeated exposure shapes these dynamics over time.

Repeated antibiotic exposure imposes strong selective pressure while allowing for surviving cells to regrow and generate new variation [4,10,32,63]. Although population bottlenecks reduce cell numbers, the surviving subpopulation can expand and introduce genetic diversity, which is then subjected to further selection during subsequent treatment cycles [10,63]. This iterative process may contribute to stepwise adaptation and increased resistance, although its extent depends on ecological and therapeutic context [4,10,32].

It may also favor resistance-conferring and secondary compensatory mutations that reduce associated fitness costs, thereby stabilizing tolerance within the population [4,63]. These multistage adaptive changes may be influenced by survival mechanisms associated with persistence in subpopulations and tolerance at the population level during earlier treatment cycles, although these phenomena are distinct [4,10,32,63]. Accordingly, repeated antibiotic exposure should be considered not only as a source of treatment failure but also an evolutionary setting that may be associated with resistance development. These dynamics must therefore be considered when designing therapeutic strategies for infections and limiting evolutionary pathways toward resistance [4,10,32,63,64].

### 5.4. Experimental Evolution Evidence

Experimental evolution studies provide important insights into the potential link between persistence and the evolution of antibiotic resistance [4,10,63,65,66,67]. Importantly, the outcomes of experimental evolution studies vary substantially depending on factors such as antibiotic type, exposure regime, population size, and environmental conditions. In these, bacterial populations were repeatedly exposed to antibiotics under controlled laboratory conditions, with defined concentrations, exposure cycles, and recovery intervals, recapitulating the processes of selection, bottlenecks, and repopulation while enabling the direct tracking of phenotypic and genetic alterations [4,10,63,65,66]. Repeated antibiotic exposure often selects first for enhanced tolerance or persistence, and is subsequently followed by the emergence of gene-conferred resistance [10,63,65,66,67]. In some experimental systems, lineages with elevated survivability during early exposure cycles have been associated with increased rates of adaptive change; however, these observations are highly dependent on experimental design and conditions, and do not universally support a direct causal relationship between persistence and resistance evolution [10,63,65,66,67]. These studies suggest that elevated tolerance or persistence can be selected under repeated exposure in certain experimental contexts, and that persistence and resistance may coexist or interact in adaptive combinations depending on environmental and selective conditions [4,63,65,66,67].

Long-term evolution-centered experiments combined with genomics have identified recurrent mutations in pathways linked to stress adaptation, metabolism, and survival, consistent with partial mechanistic overlap between persistence-associated physiology and resistance evolution [4,63,66,67]. In this sense, regulatory changes that enhance stress adaptation may also help create conditions favorable for the later evolution of resistance [4,63,67].

Together, these findings indicate that, in some experimental settings, antibiotic exposure may alter patterns of adaptation over successive treatment cycles in ways that involve persistence-associated survival, although such effects are not universally observed [4,10,63,65,66,67]. In repeated-selection environments, persister-enriched survivors preserve evolutionary continuity and re-enter subsequent rounds of selection, thereby supporting gradual adaptation [4,10,63,65,66,67]. Experimental evolutionary analysis provides important insights into the potential relationship between persistence-associated survival and the development of resistance; however, these findings are context-dependent, highly sensitive to experimental design, and do not establish a universal or directional relationship. These findings suggest that antibiotic treatment strategies must incorporate an evolutionary perspective that includes persistence formation and population dynamics, rather than focusing solely on resistance-conferring genes [4,10,63,65,66,67].

## 6. Molecular Pathways Linking Tolerance and Resistance

Traditionally, antibiotic resistance, tolerance, and persistence have been treated as distinct concepts: the former increases the MIC through heritable changes, whereas the latter reduces the rate of bacterial killing without necessarily altering the MIC [49]. However, recent studies suggest that these phenomena are not fully independent but may partially share regulatory and physiological pathways [22,32,68]. Such an overlap provides a molecular framework through which tolerance-associated survival can may be associated with processes linked to resistance emergence [22,32,41,49,68,69,70,71]. Thus, the problem of antibiotic resistance should be addressed not only in terms of resistance-related genes themselves but also through an integrated view of the molecular pathways regulating tolerance and their evolutionary interactions [22,32,41,49,68,69,70,71].

### 6.1. Overlapping Regulatory Pathways

Global stress regulators and metabolic reprogramming pathways can influence tolerance and resistance-associated processes [22,32,41,68,69]. For example, (p)ppGpp signaling can promote persistence-associated low-metabolic-rate states while also influencing survival-associated physiology and, in some contexts, mutation susceptibility [22,41,68,69]. Global regulators, such as RpoS, enhance stress adaptation and population survival under antibiotic exposure, thereby shaping the conditions facilitating long-term adaptation [32,71]. These networks coordinate short-term survival and shape the conditions for long-term adaptation, blurring the boundaries between tolerance, persistence, and resistance at the regulatory level [22,32,41,68,69,70].

### 6.2. Efflux Pumps and Cell Membrane Rearrangement

Efflux pump overexpression is a classical resistance-associated mechanism; in addition, suppressed antibiotic accumulation can also contribute to tolerance-like survival by delaying bacterial killing [49,70,71]. Similarly, alterations in membrane permeability, porin expression, or envelope composition can decrease antibiotic influx and support temporary survival while also providing a basis for the later development of more stable resistance phenotypes [70,71]. Thus, efflux regulation and membrane remodeling lie at a crucial interface between tolerance and resistance [49,70,71,72].

### 6.3. Global Stress Regulators and Adaptive Pathways

Global stress regulators are also closely associated with persistence and resistance. The SOS response can increase mutation frequency through DNA repair and error-prone replication pathways while simultaneously contributing to survival under antibiotic stress [32,41,69]. More broadly, oxidative stress responses, metabolic regulators, and signaling networks can coordinate transient survivability and conditions that permit later adaptive change by modulating cell-level physiology [22,32,41,68,69,70,71]. This overlap suggests that tolerance and resistance can be viewed as partially continuous at the regulatory architecture and evolutionary levels [32,49,68]. Under recurrent antibiotic exposure, mutations or regulatory variations within these overlapping networks may later be selected and stabilized as resistance determinants, ultimately producing conspicuous resistance phenotypes, such as increased MICs [32,41,49,68,69,70,71].

## 7. Clinical Implications

### 7.1. Chronic and Recurrent Infections

The clinical significance of persister cells is particularly evident with chronic and recurrent infections, which often fail to resolve completely despite apparent antibiotic susceptibility and frequent relapse post-treatment [43,53,57,73]. Such a pattern is difficult to explain solely by classical resistance mechanisms, and the survival of persister cells during therapy is considered a contributive factor [53,57,73]. Persister cells remain in low-metabolic or slow-growing states during antibiotic exposure, evade killing, and can resume proliferation after treatment ends, thereby contributing to relapse [43,53,57,73].

Chronic infection-associated environments provide conditions favorable for persister formation. In particular, biofilms generate pronounced physiological heterogeneity through nutrient and O_2_ gradients, immune-associated stress, crowding, and accumulation of metabolic byproducts [74,75,76]. Such environments promote low-metabolic, antibiotic-tolerant, and persister-like subpopulations during exposure [74,75,76]. This phenomenon is particularly relevant in cystic fibrosis lung infections, chronic urinary tract infections, osteomyelitis, chronic wound infections, and prosthetic joint or device-associated infections, all of which are difficult to eradicate despite repeated antimicrobial therapy [53,57,75,76,77].

The problem of recurrent infections extends beyond elevated clinical burden and carries evolutionary implications. Repeated antibiotic treatment eliminates susceptible cells while leaving behind persister-associated survivors. During subsequent repopulation, adaptive changes may occur during subsequent therapeutic phases [43,57,73]. Thus, what begins as phenotypic tolerance can, over time, facilitate the emergence of gene-level resistance. Therefore, chronic and recurrent infections provide a major clinical setting in which persistence and resistance-associated evolution can interact [43,53,57,73].

These dynamics are especially pronounced in immunocompromised patients and in individuals requiring prolonged antibiotic therapy, where incomplete host clearance allows persister-associated survival over extended periods [43,53,73]. Recurrent antibiotic exposure can reinforce cycles of survival, regrowth, and adaptation, contributing to prolonged infection and conditions favorable for resistance accumulation and limited treatment options [43,53,57,73].

### 7.2. Treatment Failure and Relapse

Failure of antibiotic treatment has traditionally been attributed to resistant bacteria, but persistence has gained increasing attention because clinical failure and relapse can occur even when no gene-level resistance is identified [49,54,73,78]. Persister cells can survive antibiotic exposure and may contribute to incomplete eradication of infection and subsequent relapse [49,54,73,78], which can often involve the same strain or closely related progeny, suggesting that treatment-surviving cells contribute to infection reinitiation [49,54,73,78].

A prominent feature of persistence-associated therapeutic failure is the discrepancy between laboratory susceptibility and clinical outcomes [49,79,80,81]. Although MIC testing may indicate susceptibility, phenotypic tolerance can permit survival during treatment [49,78,79,80,81]. Because persister cells reduce killing efficiency without altering MIC, MIC-centered diagnostic systems may fail to capture this form of recalcitrance [49,78,79,80,81]. Consequently, even intensive regimens may leave behind a small fraction of slow-growing or low-metabolism survivors [49,54,82].

In addition, relapse has crucial evolutionary implications. Persister-associated survivors can serve as the parent population for regrowth after initial treatment, and during this phase, genetic changes may arise and be selected during subsequent therapy [54,73,83]. Thus, persistence is not merely a result of delayed clearance but may function as an intermediate survival-associated state that can, under certain conditions, contribute to resistance evolution [54,73,83]. Recurrent relapses may create conditions that are favorable for stepwise adaptive evolution under repetitive treatment [54,73,83].

Pharmacokinetic and pharmacodynamic features are also closely linked to persister survival. Uneven tissue penetration, fluctuating levels of localized drug exposure, or a rapid reduction in concentrations after antibiotic discontinuation can favor survival of tolerant cells and permit regrowth [79,82,83]. These conditions can reinforce the cycle of survival, regrowth, and reselection, thereby increasing the possibility of resistance emergence [79,82,83].

Clinically, therapeutic failure and relapse may contribute to prolonged hospitalization, repeated antibiotic exposure, increased healthcare costs, and a greater risk of complications [54,73]. These problems are especially pronounced in immunocompromised patients and those with chronic underlying diseases, in whom incomplete clearance may permit long-term survival of persister-associated subpopulations [54,73]. Thus, treatment strategies that ignore persistence may only transiently suppress microbes while inducing conditions favorable for relapse and resistance evolution [49,54,73,78,79,80,81,82,83]. Therapeutic failure and recurrence, therefore, provide a clear clinical example of how persistence can be connected directly to evolutionary processes and must be considered alongside resistance-conferring genes when designing therapeutic strategies [49,54,73,78,79,80,81,82,83].

### 7.3. Implications for Antibiotic Stewardship

Traditionally, strategies for antibiotic stewardship have focused on minimizing unnecessary prescriptions and inappropriate use of broad-spectrum antibiotics, optimizing dose and treatment duration [79,82]. However, when persistence is considered, stewardship requires a broader framework that considers resistance-associated selection, tolerance, persistence, and the downstream evolutionary consequences [31,32,83].

As persister-associated survivors can tolerate antibiotics, prevent eradication, and seed regrowth during repeated exposure scenarios, stewardship strategies must consider not only how to control resistant bacteria but also how treatment design can limit the survival of persister-associated subpopulations [31,32,83]. If drug exposure fluctuates substantially or local concentrations are ineffective, cycles of survival, regrowth, and reselection may be reinforced, thereby accelerating evolutionary adaptation [79,82,83]. Accordingly, highly aggressive short-duration strategies (shorter-than-standard or insufficient treatment courses depending on clinical context) may not always be evolutionarily optimal if they allow a small fraction of persister-associated survivors. Strategies that intensify selection conditions without considering persistence dynamics may therefore increase the potential risk of resistance-associated evolution [31,79,82,83]. This phenomenon highlights the need to redesign antibiotic therapy from an evolutionary perspective. In addition, adjunct approaches that activate bacterial metabolism or directly target persister-associated physiology may enhance the eradication of whole populations when combined with conventional antibiotics [32,84,85].

Another crucial component of stewardship is optimizing treatment duration. Excessively prolonged therapy can sustain selection conditions and promote adaptive evolution, whereas too short a treatment may leave persister-associated survivors and increase relapse risk [79,82,83]. Treatment duration should therefore be optimized while considering persistence, regrowth risk, and population dynamics rather than solely conventional [79,82,83]. This effect requires the integration of clinical evidence with pharmacokinetics/pharmacodynamics data, experimental evolutionary findings, and evolutionary modeling [31,79,82,83].

From such a perspective, stewardship extends beyond short-term infection control toward limiting the evolution of resistance [31,32,83]. Antibiotics are not only therapeutic agents, but also developmental forces, and stewardship can therefore be viewed as a strategy that seeks to shape microbial evolutionary outcomes while maximizing therapeutic efficacy [31,32,79,82,83,84]. Such an integrated approach may improve treatment outcomes while reducing the risk of persistence-mediated resistance evolution [31,32,79,82,83,84,85].

To enhance the clinical applicability of these concepts, several practical antimicrobial stewardship strategies can be considered. First, optimizing antibiotic dosing and duration based on pharmacokinetic and pharmacodynamic principles may reduce unnecessary exposure while maintaining therapeutic efficacy [86]. Second, minimizing sublethal antibiotic exposure—such as through appropriate dosing regimens—may help limit conditions that allow survival of tolerant or persistent subpopulations [10]. Third, combination therapies targeting both actively growing and less metabolically active bacterial subpopulations may improve treatment outcomes in certain contexts, particularly in chronic or biofilm-associated infections [87]. Finally, the use of rapid diagnostics and individualized treatment strategies may contribute to reducing inappropriate antimicrobial use [88]. Importantly, these approaches should be interpreted as complementary to established evidence-based stewardship practices, as direct clinical evidence specifically linking persistence-targeted strategies to reduced antimicrobial resistance remains limited.

## 8. Therapeutic Strategies Targeting Persister Cells

### 8.1. Antipersister Compounds

As persister cells contribute to the failure of antibiotic treatment and later resistance evolution, antipersister compounds and adjunct strategies designed to sensitize or eliminate these cells are being explored as potential therapeutic approaches, although their clinical applicability remains to be fully established [84,89,90,91,92,93,94]. Conventional antibiotics primarily target physiological processes such as cell wall synthesis, protein translation, and DNA replication, and therefore often show limited efficacy against low-metabolism persister cells [89,90,91,94]. Thus, antipersister approaches aim to either directly kill dormant or low-metabolism persister cells or to disrupt the physiological states that protect from conventional antibiotics [84,89,90,91,92,93,94].

Antipersister-based strategies can be broadly divided into direct killing and persister-sensitizing [90,91,94]. The former often target functions that remain vulnerable even in low-metabolism states, such as membrane integrity, membrane potential, or ion homeostasis [84,90,92,95]. The latter attempts to weaken persister protection by interfering with stress-response pathways, TA-associated programs, or metabolic control mechanisms [89,91,93,94,96]. Host-derived antimicrobial peptides and a few natural compounds exhibit antipersister activity in preclinical settings, particularly through membrane disruption or related impacts [84,92]. Most of these strategies may be most effective when combined with antibiotics rather than as monotherapies [84,90,91,92,94].

From an evolutionary perspective, effective persister elimination may help disrupt the cycle of survival, regrowth, and reselection that underlies subsequent resistance emergence [89,91,94]. By reducing post-treatment survival and regrowth opportunities, antipersister strategies may improve short-term eradication while also lowering opportunities for persistence-mediated long-term evolution [89,91,94].

However, important challenges remain. As persister cells are reversible physiological states rather than a single genotype, selective targeting is difficult; host toxicity must be considered [89,93,94,96]. In addition, any new antipersister pressure may introduce new selective dynamics, making evolutionary evaluation an essential part of therapeutic design [89,91,94,96].

Overall, antipersister compounds represent a promising adjunct strategy for weakening the link between persistence and resistance evolution. The development of novel antimicrobials may therefore need to extend beyond bactericidal activity toward approaches that also limit persistence-associated survival and downstream evolutionary outcomes [84,89,90,91,92,93,94,96]. From a clinical perspective, these approaches may be most relevant as adjunctive therapies, although their safety and efficacy in humans remain to be established.

### 8.2. Metabolic Activation Approaches

The defining physiological feature of persister cells is their low-metabolic, low-energy state, which renders many bactericidal antibiotics that depend on active metabolism and growth-associated processes almost ineffective [78,89,91,97]. Hence, one proposed therapeutic strategy is to transiently reactivate persister metabolism, thereby increasing their susceptibility to active physiology-dependent antibiotics [78,89,91,97,98], a concept that is commonly referred to as a wake-and-kill strategy [78,89,91,98].

Metabolic activation-based strategies may promote transitions of persisters toward more growth-competent states by stimulating selected metabolic pathways or increasing energy production [78,91,97,98]. In some systems, the provision of specific carbon sources or metabolic stimuli enhances ATP production, membrane potential, or related physiological activities, thereby improving antibiotic uptake and efficacy [78,91,98]. This phenomenon is particularly relevant for aminoglycosides, whose uptake depends on membrane potential and metabolic activity, and which can enhance the efficacy of persister-enriched populations when combined with metabolic stimulation [22,78,91,98].

A related approach is to modulate intracellular signaling systems that help maintain low-activity states [22,78,89,91,97,99]. For example, (p)ppGpp-associated stress regulation and energy-sensing networks are putative targets for shifting cells away from protected survival states toward antibiotic-sensitive physiology [22,91,99,100]. Unlike direct antipersister killing, this strategy aims to restore or enhance antibiotic activity under certain conditions by changing bacterial physiology [78,89,91].

From an evolutionary perspective, successful wake-and-kill strategies may help suppress the survival–regrowth–reselection cycle that allows persisters to seed later resistance emergence [78,89,91,97]. Metabolic activation may improve short-term eradication by lowering opportunities for post-treatment survival and regrowth, as well as the evolution of persistence-mediated resistance [78,89,91,97].

However, this strategy also requires caution, as metabolic reactivation can increase cell growth and activity; it may expand adaptive opportunities if antibiotic exposure is insufficient or poorly timed [78,91,97]. Wake-and-kill approaches must therefore be coordinated with adequate antibiotic concentrations, appropriate timing, PK/PD behavior, and population dynamics [78,91,97].

Overall, metabolism-based activation is a promising strategy for restoring antibiotic susceptibility in persister-enriched populations. Future innovation should focus on clinically feasible designs that maximize synergy with existing antibiotics while minimizing unwanted evolutionary responses [78,89,91,97,98]. Careful synchronization with antibiotic exposure is likely required, as inappropriate activation could potentially enhance bacterial growth or adaptive capacity.

### 8.3. Combination Therapies

Among strategies for improving the eradication of persister-enriched populations, combination therapy represents a potential approach in certain clinical contexts [4,31,44,92,97,101,102]. Single-antibiotic therapy often acts preferentially on bacteria of particular physiological states and may therefore fail to eliminate hypometabolic or dormant persister-associated subpopulations [92,97,102]. By contrast, well-designed combinations may simultaneously target subpopulations that vary in physiological states, thereby increasing the probability of whole-population clearance [4,31,44,92,97,101,102].

Such strategies can be broadly grouped into regimens that pair conventional antibiotics with antipersister agents, regimens that combine wake-and-kill metabolic stimulation with antibiotics, and regimens that apply mechanistically distinct antibiotics in parallel [92,97,101,102]. In the first case, one drug targets active cells, whereas the second agent either kills low-metabolism persisters or weakens physiological protections that allow their survival [92,97,102]. In the second, metabolic activation is employed to increase antibiotic susceptibility before transient killing [97,101,102]. In the third, simultaneous suppression of multiple essential pathways may reduce survival potential when the regimen is mechanistically compatible and pharmacologically coordinated [31,92,101].

Another major advantage is its potential to suppress resistance-associated evolution [4,31,44]. Compared with monotherapy, appropriately designed combinations may increase the evolutionary barrier to resistance by requiring multiple compatible adaptations for survival [4,31,44]. This phenomenon can reduce the number of accessible adaptive trajectories and weaken the survival–regrowth–reselection cycle associated with persister-mediated resistance emergence [4,31,44].

However, combination therapy requires careful design. Antagonism can reduce efficacy, and poorly designed regimens may generate unnecessary selective pressure or open unanticipated adaptive pathways [4,31,44,101,102]. Drug toxicity, pharmacokinetic compatibility, and patient adherence also remain crucial practical constraints [97,101,102]. Thus, this strategy should not be viewed as a simple addition of drugs but as an optimized strategy informed by bacterial physiology, population dynamics, and evolutionary risk [4,31,44,92,97,101,102].

From an evolutionary perspective, combination therapy can be framed as a form of selection management [4,31,44]. The goal is to design combinations that weaken the survival niche of persister-associated subpopulations, restrict accessible adaptive pathways, and lower the probability of resistance inheritance [4,31,44,101,102]. In this sense, combination therapy represents a strategic treatment framework that accounts for physiological heterogeneity, population dynamics, and long-term evolutionary outcomes rather than focusing on only a single target [4,31,44,92,97,101,102]. Rational design based on pharmacodynamic compatibility and resistance risk will be essential for clinical implementation [87].

### 8.4. Phage and Alternative Therapeutics

Because persister cells often evade the mechanisms of action of conventional antibiotics, alternative therapeutic strategies with different modes of action have attracted increasing interest [85,103,104,105,106]. Among these, bacteriophage-based therapy is a prominent approach: bacteriophages infect specific bacteria, replicate within them, and ultimately lyse the host cells [85,103,104,105]. Phages offer high host specificity and may retain activity in settings such as biofilms, where conventional antibiotics are less effective [85,103,104,105,106,107,108,109].

Phage therapy has been proposed to offer several potential advantages, although these remain context-dependent for the persistence problem. Because phages initiate infection through the recognition of bacterial surface receptors, they may access subpopulations that are poorly targeted by conventional antibiotics [85,103,104,105,106,107,108,109]. In addition, phages can amplify locally through replication in susceptible bacteria, making them less dependent on externally maintained drug concentration than conventional antibiotics [85,103,104,105]. Phage–antibiotic combinations can, in some cases, produce synergistic impacts, although it depends on the particular phage, bacterial strain, and treatment context [104,107,108,109].

Beyond phages, strategies under investigation include antimicrobial peptides, immunomodulatory approaches, and CRISPR-based antibacterial systems [85,103,105,106,110]. Antimicrobial peptides can disrupt bacterial membranes and may therefore retain activity against low-metabolism persister-associated cells [85,103,105,106]. CRISPR-based systems are being developed as targeted platforms capable of selectively disrupting resistance determinants or sensitizing defined bacterial genotypes [85,103,110].

From an evolutionary perspective, these alternatives can reshape selective landscapes in ways that vary from conventional antibiotic-only treatment [85,103,104,105,106,107,108,109,110]. They may weaken the survival–regrowth–reselection cycle associated with persistence by disrupting bacterial survival through distinct mechanisms [103,106,107,108,109,110]. However, they remain susceptible to counter-adaptation in bacteria, including phage receptor modification, CRISPR-mediated defenses, and envelope remodeling [104,106,107,108,109,110]. Thus, these approaches are likely to be most effective when integrated into combination or rotation-based treatment frameworks rather than as monotherapies [104,107,108,109,110].

Bacteriophages and alternative therapeutics represent promising complementary approaches for targeting persistence and evolutionary pathways that may contribute to resistance. Future treatment paradigms may require the integrated use of antibiotics with diverse biological tools rather than reliance on antibiotics alone [85,103,104,105,106,107,108,109,110]. Importantly, many of these approaches remain in preclinical or early application stages, and their clinical efficacy, safety, and potential unintended consequences require further validation in well-controlled studies.

## 9. Limitations and Future Perspectives

Although understanding the relationship between persister cells and the evolution of antibiotic resistance has expanded rapidly, major questions remain. Addressing these requires a multilevel framework spanning molecular mechanisms, population dynamics, and clinical reality [111,112,113,114,115,116,117]. Single-cell analysis, systems biology, evolutionary modeling, and clinically integrated longitudinal studies can be particularly critical for clarifying the contribution of persistence to the evolution of resistance [111,112,113,114,115,116,117]. These limitations should be considered when interpreting the proposed links between persistence and resistance throughout this review.

### 9.1. Limitations of the Current Literature

Despite growing interest in bacterial persistence and its proposed relationship to antibiotic resistance, several important limitations of the current literature should be acknowledged. First, much of the available evidence is derived from in vitro experimental systems, which may not fully capture the complexity of in vivo infections. Factors such as host immunity, tissue-specific microenvironments, spatial heterogeneity, and pharmacokinetic variability can substantially influence bacterial survival and treatment outcomes. Second, persistence is highly heterogeneous across bacterial species, strains, and ecological contexts. Mechanisms implicated in persistence in one organism may not be directly generalizable to others, and no single model system fully represents the diversity of persistence-associated biology. Third, although experimental evolution studies provide valuable mechanistic insights, their findings remain highly dependent on laboratory conditions, including antibiotic type, exposure regime, and population structure. These constraints limit direct extrapolation to clinical infections. Finally, longitudinal clinical studies directly linking persistence to treatment failure, relapse, and subsequent resistance emergence remain scarce. As a result, the real-world contribution of persistence to resistance evolution in patients is still difficult to define with certainty. Taken together, these limitations indicate that the relationship between persistence and resistance should be interpreted cautiously and in a context-dependent manner. Future progress will require integrative approaches combining experimental, clinical, and systems-level investigations.

### 9.2. Advances in Single-Cell Analysis Technology

Because persister cells represent a rare subpopulation, traditional population-averaged methods are insufficient to characterize them [111,116]. Recent advances in single-cell transcriptomics, microfluidic time-lapse imaging, and fluorescent reporter systems improve the possibility of tracking physiological state transitions, survival, awakening, and lineage-specific outcomes in real time [111,116]. These technologies are essential for directly testing how persistence, resuscitation, and later resistance emergence are connected at the single-cell level [111,115,116].

### 9.3. Systems Biology Approaches

Persister formation is best viewed as an emergent system-level property arising from interactions between stress-response, metabolic, and regulatory networks rather than a single gene or pathway [112,114]. Future progress would therefore depend on integrating transcriptomic, proteomic, metabolomic, and modeling approaches to reconstruct the regulatory architecture of persistence [112,114,116]. Such system-level models may help predict how short-term physiological survival influences longer-term adaptive trajectories [112,114].

### 9.4. Designing Evolution-Based Therapeutic Strategies

As antibiotics impose robust selection through which persistence promotes survival, future therapies should be designed not only to reduce bacterial burden but also to limit adaptive evolutionary opportunities [113,114]. This method includes optimizing the timing, intensity, and combination structure of treatment to inhibit population recovery under antibiotic exposure and post-treatment regrowth [113,114]. Therapeutic strategies informed by evolutionary modeling and experimental data may therefore help slow or redirect resistance evolution [113,114].

### 9.5. Integration of Clinical Data and Experimental Evolution

Although laboratory evolution studies provide critical mechanistic insights, the clinical environment is shaped by host immunity, pharmacokinetic variability, tissue microenvironments, and patient-to-patient heterogeneity [113,117]. Future progress would require linking experimental models with collections of longitudinal clinical isolates, whole-genome sequencing, and treatment-history metadata [113,114,117]. Such integration is essential for estimating the real-world contribution of persistence to resistance evolution in patients [113,117].

### 9.6. Exploring the Evolutionary Origins of Persistence

Finally, an important unresolved question is whether persistence is primarily a byproduct of stress physiology or an evolved bet-hedging strategy maintained by fluctuating environmental conditions [112,118]. If persistence does reflect an evolutionarily selected survival strategy, then it may predate modern antibiotic use and represent a more general adaptive response to environmental uncertainty [112,114,118].

## 10. Conclusions

Antibiotic resistance is not merely a phenomenon explained by the acquisition of specific genes or the accumulation of mutations; it is a dynamic biological process shaped by repeated antibiotic exposure and differential survival. This review reexamines the role of persister cells within this context. Persister cells do not possess the genetic basis for resistance; instead, they represent a population that can prevent population extinction during antibiotic exposure and maintain a foundation for survival, thereby potentially expanding the opportunity for resistance evolution under certain conditions. Importantly, persistence is not a universal prerequisite for resistance evolution, and multiple independent evolutionary pathways can lead to the emergence of antibiotic resistance.

At the molecular level, the TA system (p)ppGpp-mediated stringent response, energy metabolism suppression, ribosome regulation, and global stress-response networks switch cells to a low-metabolism, low-growth state, inducing transient antibiotic tolerance or persistence. Such phenotypic heterogeneity allows a fraction of cells to survive antibiotic treatment, initiating a survival–regrowth–reselection cycle upon repeated exposure. This process may facilitate genetic changes under certain conditions, potentially contributing to the gradual transition from initial tolerance into genetic resistance.

Clinically, persister cells are considered one of several contributing factors to chronic and recurrent infections and treatment failure, while simultaneously providing a practical foundation for the evolution of resistance in repeated treatment environments. This observation implies that persistence should be understood not merely as a therapeutic obstacle but as a precursor to evolution. Therefore, solving the problem of antibiotic resistance requires an integrated consideration of the genetic mechanisms of resistance and the molecular networks that regulate tolerance and population dynamics.

From a therapeutic strategy perspective, antipersister compounds, metabolic activation approaches, combination therapies, phages, and alternative strategies present promise for simultaneously targeting persistence and resistance evolution. It is particularly crucial to reinterpret antibiotic treatment not merely as a bactericidal but as a selection-pressure management strategy that modulates the evolutionary pathways of bacterial populations. It can be considered an essential condition for achieving both short-term treatment success and long-term suppression of resistance.

In conclusion, persister cells are not a concept opposed to antibiotic resistance but rather a key factor shaping its evolutionary trajectory. Understanding and targeting persistence can serve as a strategic starting point to fundamentally suppress the emergence and spread of antibiotic resistance, going beyond mere improvements in treatment efficacy. Future research should aim to interpret persistence and the evolution of resistance within a unified evolutionary framework and develop treatment and management strategies based on this understanding. From a clinical perspective, integrating persistence-aware strategies into antimicrobial stewardship may offer additional opportunities to optimize treatment, although further clinical validation is required. By explicitly integrating mechanistic, evolutionary, and clinical perspectives while addressing current limitations and uncertainties, this review provides a framework that extends beyond existing descriptive accounts of bacterial persistence.

## Figures and Tables

**Table 1:** Comparative summary of proposed molecular mechanisms associated with bacterial persister formation.

Mechanism	Proposed Role in Persistence	Experimental Support	Representative Organisms	Major Limitations /Controversies
Toxin–antitoxin (TA) systems	Induction of growth arrest and translational inhibition leading to phenotypic heterogeneity and survival of a subpopulation	Moderate but inconsistent; strain- and condition-dependent	*Escherichia coli*, *Staphylococcus aureus*, *Mycobacterium tuberculosis*	Contribution remains controversial; some studies report minimal or no effect; not universally required for persistence
Stringent response ((p)ppGpp)	Global transcriptional and metabolic reprogramming; suppression of growth-associated processes; promotion of low-activity states	Moderate to strong, but context-dependent	*E. coli*, *Bacillus* spp., *M. tuberculosis*	Role varies across species and environmental conditions; not sufficient alone to explain persistence
ATP depletion/metabolic slowdown	Reduction in cellular energy levels and biosynthetic activity, decreasing susceptibility to antibiotics targeting active processes	Strong association observed; causal role remains debated	*E. coli*, *S. aureus*	Unclear whether ATP depletion is a driver or consequence of persistence; system-dependent effects
Ribosome modulation/translational arrest	Decreased translational activity and entry into dormant-like states that reduce antibiotic sensitivity	Moderate	*E. coli*, mycobacteria	Frequently associated with persistence but not universally sufficient; mechanistic details remain incomplete
Stress response networks (e.g., SOS response, RpoS pathways)	Integration of environmental stress signals; induction of survival programs and physiological heterogeneity	Moderate, highly context-dependent	Multiple bacterial species	Broad and overlapping regulatory systems; difficult to attribute specific causal roles; limited mechanistic specificity

## Data Availability

No new data were created or analyzed in this study. Data sharing is not applicable to this article.

## Abbreviations

The following abbreviations are used in this manuscript:

MIC	Minimum inhibitory concentration
TA	Toxin–antitoxin
